# PERMA model vs. integrative-behavioral couple therapy for fertility problems: A randomized clinical trial protocol

**DOI:** 10.18502/ijrm.v19i12.10061

**Published:** 2022-01-12

**Authors:** Mahsa Sadeghi, Tahmineh Farajkhoda, Mahdi Khanabadi, Maryam Eftekhar

**Affiliations:** ^1^Student Research Committee, School of Nursing and Midwifery, Shahid Sadoughi University of Medical Sciences, Yazd, Iran.; ^2^Research Center for Nursing and Midwifery Care, Shahid Sadoughi University of Medical Sciences, Yazd, Iran.; ^3^Department of Counseling, Allameh Tabataba'i Univercity, Tehran, Iran.; ^4^Abortion Research Center, Yazd Reproductive Sciences Institute, Shahid Sadoughi University of Medical Sciences, Yazd, Iran.; ^5^Research and Clinical Center for Infertility, Yazd Reproductive Sciences Institute, Shahid Sadoughi University of Medical Sciences, Yazd, Iran.

**Keywords:** Online, Face-to-face, Infertility, Integrative-behavioral couple therapy, PERMA model, RCT, Positive psychology, Protocol study, COVID-19.

## Abstract

**Background:**

Psychological interventions may reduce fertility problems. Positive emotion, engagement, meaning, positive relationship and accomplishment (PERMA) is a cognitive intervention and integrative-behavioral couple therapy (IBCT) is a behavioral intervention. Appropriate mental interventions are important in infertility treatment.

**Objective:**

To investigate the effect of the PERMA model vs. IBCT in reducing the fertility problems of couples.

**Materials and Methods:**

The content of the interventions was developed and adjusted based on a literature review and the opinions of experts. In this three-arm parallel randomized clinical trial, 42 couples undergoing infertility treatment will be allocated randomly into three groups (n = 14 couples/each). Group 1 will receive the PERMA intervention, group 2 will receive the IBCT intervention, and group 3 as the control group will receive an infertility treatment training program intervention. The primary outcome will be the fertility problems, which will be measured by completing a fertility problem inventory at baseline, as well as in the 5 ${}^{\text{th}}$ and 9 ${}^{\text{th}}$ wk as a follow-up. Another primary outcome, satisfaction with the intervention, will also be assessed in the 5 ${}^{\text{th}}$ and 9 ${}^{\text{th}}$ wk. The secondry outcome will be a positive pregnancy test at wk 12. The interventions will be conducted through a combination of face-to-face and online via WhatsApp.

**Conclusion:**

This study will assess social, sexual, and parenthood concerns. A combination of online and face-to-face interventions will be appropriate given the COVID-19 pandemic. Couple's counseling may provide better counseling outcomes for fertility problems in comparison with group counseling. This study will try to optimize resilience during infertility treatment through learning better relationship and problem-solving skills, and may have an indirect impact on pregnancy rate, burden of infertility, and costs of treatment due to increased effectiveness.

## 1. Introduction 

Infertility is defined as the absence of pregnancy following a year of regular sexual intercourse (without the use of contraception) (1). The fertility problem inventory (FPI) measures perceived infertility-related stress and specific domains of patient concern. Worldwide prevalence of primary infertility is about 12-15%. According to Sun and colleagues, from 1990 to 2017, the prevalence of primary infertility increased to 14.9% in women and 8.2% in men (2). In another study, infertility prevalence was reported as nearly 13.2% in Iran (3).

Infertility treatments, from medical monitoring to fertilization procedures, impose a severe physical and psychological burden on women and their husbands (4). Women undergoing infertility treatments report higher levels of stress, anxiety, and depression, which affect their quality of life, marital relationships, and sexual functioning. They may experience various psychological problems, such as low self-esteem, anger, sadness, anxiety, and jealousy towards other couples who have children. Fear of childbirth may develop in women who have more extended anxiety during infertility treatment, which may lead to more pregnancy complications and operative deliveries in comparison with women who conceive naturally, because of the effect of fear on unsuitable cervical ripening and preparation for vaginal delivery (5, 6).

For these reasons psychological counseling and interventions are necessary. Psychological intervention can relieve the patients' stress, depression, and fatigue, facilitate marital intimacy, and promote sexual satisfaction (7). Due to the outbreak of COVID-19 and the necessity to comply with social distancing, mental disorders in this group have increased. Nowadays, using new online telecommunication software instead of face-to-face counseling can reduce the psychological problems of couples with infertility (8).

Different infertility treatment problems, such as costs, complexities and complications of medical treatments can activate body stressors. Cortisol has a negative effect on infertility (9). In contrast, having a positive mood is beneficial on cardiovascular function including heart rate and blood pressure, and also on the immune system, which can be associated with better fertility outcomes (9, 10).

Psychological counseling interventions may be effective in decreasing fertility problems by providing skills for couples to manage their stress during infertility treatment (11). One of these cognitive interventions that can be used is a positive-oriented intervention which includes elements focusing on: 1) positive emotions; 2) engagement; 3) relationship; 4) meaning; and 5) accomplishment. This is called the PERMA model and it is based on Seligman's PERMA theory of well-being (12-15). Behavioral approaches can also be used for infertile couples' therapy. One such model is integrative-behavioral couple therapy (IBCT) (16). This approach is primarily focused on strategies to promote emotional acceptance in relationships. These strategies focus on feeling empathy, showing tolerance for problems, gaining problem-solving skills and changing the couple's behavior (17). Many couples undergoing infertility treatment are not fully aware of the treatment process and they complain about this issue. This informational gap might lead to anxiety. Provision of information regarding infertility treatment methods can help to reduce anxiety among couples undergoing infertility treatment (18).

Although some studies have been conducted regarding fertility problems (7, 19), our approach is novel in comparing three different counseling methods, including a positive-oriented therapy (PERMA, focusing on increasing good mood), couple's therapy (IBCT, focusing on enhancement of the couple ${}^{{}^{'}}$ s relationship), and an infertility treatment training program, using new online telecommunication software, plus face-to-face counseling. Since cognitive and behavioral approaches are both common for psychological counseling in cases of infertility treatment (11), both were included in the study.

This study will aim to compare the effectiveness of the PERMA model vs. IBCT in reducing the fertility problems of couples undergoing infertility treatment.

## 2. Materials and Methods

This randomized clinical trial will be conducted in 2021. The primary objects of study will be a comparison of the effectiveness of the PERMA model vs. IBCT on reducing the fertility problems of couples undergoing infertility treatment, and satisfaction with the interventional methods.

### Objects

**Figure 1:**
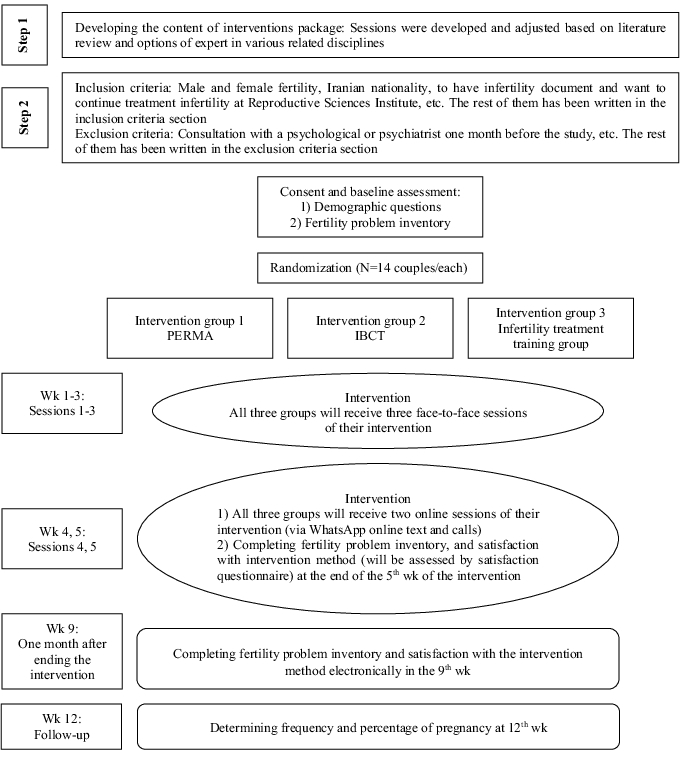
Study protocol. PERMA: Positive emotion, engagement, meaning, positive relationship and accomplishment, IBCT: Integrative-behavioral couple therapy.

The primary object is determining and comparing the mean scores of fertility problems in women, men and couples at the baseline of the intervention, at the end of the 5 ${}^{\text{th}}$ wk of the intervention, and one month after ending the intervention (in the 9 ${}^{\text{th}}$ wk) in the three study groups. The other primary outcome will be the mean satisfaction scores of the interventional method in women, men and couples at the end of the 5 ${}^{\text{th}}$ wk of the intervention and one month after the intervention (in the 9 ${}^{\text{th}}$ wk) in the three study groups. The secondary outcomes will be the frequency and percentage of pregnancy at the time of follow-up (in the 12 ${}^{\text{th}}$ wk) in the three study groups (Figure 1).

### Study design 

There are two steps in this study, including developing and adjusting the content of the intervention package in step one and conducting the intervention as a randomized clinical trial in step two. The study design is shown in figure 1.

#### Step one: Developing the content of interventions' packages

Since details of counseling sessions in PERMA, IBCT, and infertility treatment training program interventions have not been reported in previous studies, especially in treating fertility problems (19, 20), the authors of the present study needed to develop and adjust a package for the content of each session for each of the three intervention groups.

Therefore, the intervention package, including the content of the sessions, the couple's responsibilities, and their homework assignments, was developed and adjusted based on a literature review (14, 21, 22) and agreement of experts in related disciplines (including reproductive health specialists, obstetricians and gynecologists, infertility fellows, a couple's therapist, a midwife and a psychologist in the Yazd Reproductive Sciences Institute). A summary of the intervention sessions is shown for the three intervention groups in tables I to III.

#### Step two: Intervention

The first group will receive the PERMA model intervention, the second group will receive the IBCT intervention, and the third group will receive an infertility treatment training program (the control group).

### Participants and setting

Participants will be couples undergoing infertility treatment at the Yazd Reproductive Sciences Institute, Yazd, Iran. This setting provides various medical and psychological facilities for couples undergoing infertility treatment. Recruitment will take place at the Yazd Reproductive Sciences Institute by the first and fourth researchers who will assess the eligibility criteria. Participants data will be kept confidential in this study.

### Inclusion criteria

The inclusion criteria will be: Iranian citizens; who are literate; have at least one smartphone and are Internet literate; have an infertility record in Yazd Reproductive Sciences Institute, Yazd, Iran; are an assisted reproductive technology (ART) candidate; and want to continue treatment at the Reproductive Sciences Institute.

### Exclusion criteria

The exclusion criteria will be: having severe fertility problems based on the FPI; having divorce documents in the courts; being a smoker or drug abuser; taking drugs for systemic diseases (except for infertility treatment drugs) according to the medical records available in the research institute; needing surgery (except for infertility surgeries); undergoing psychotherapy; having severe depression or anxiety; consulting a psychologist or psychiatrist one month or less before the study; being health care personnel; studying medical sciences, psychology, or counseling; changing residence location during the sampling period; participating in similar studies concurrently; having a problem that leads to sadness, anxiety, and depression, like the death of family members, etc. in the last two months; having a systemic disease; or being pregnant.

### Sampling and sample size

The estimated sample size is 84 husbands and wives (14 couples in each group) based on a previous study (23) and considering α = 0.05, β = 90%, power = 80% and σ = 2.5, and 15% attrition rate. The statistical formula used for sample size determination was n = 2 (z 1- α / 2 + z 1 - β )^2^ σ ^2^ / (µ1-µ2)^2^.

In this three-arm parallel randomized clinical trial, two intervention groups (the PERMA model and IBCT) and a control group (infertility treatment training program) will be studied. 42 couples will be allocated into the three groups randomly (14 in each). Written and verbal informed consent will be obtained from husbands and wives separately.

### Randomization

After the fourth author confirms the eligibility of the couples using the eligibility criteria, the method of generating the random allocation sequence (e.g., computer-generated random numbers) will be conducted using www.randomization.com by the first author. Each couple will receive a number from 1 to 42 to randomly allocate them into the A, B, or C group based on the randomization list.

### Blinding

No blinding will be performed in this study because couples will be aware of their intervention method.

### Intervention

Interventions will be conducted by the first author, who has a certificate in midwifery counseling, under the supervision of the second and third authors. All three interventions groups will receive five weekly intervention sessions (one session per wk) in a combination of threeone-hrface-to-face sessions (which they will attend at the Yazd Reproductive Sciences Institute in order to be visited by physicians, receive their drugs or do their medical tests, based on routine care at the Yazd Reproductive Sciences Institute) and two online sessions via telecommunication software (WhatsApp sessions showing clips and films, presenting Powerpoints, and having online video or call chats). All of the face-to-face intervention sessions will be held in the same place (in the Yazd Reproductive Sciences Institute) with similar counselors in the three groups. The same counselor will assess the couple's homework assignments in the three groups. A daily reminder will be sent to couples via short text messages or through WhatsApp.

#### Intervention group 1

The first intervention group (PERMA model) will receive counseling with the content of positive-oriented psychology (14). Positive psychology focuses on mental, individual, and social areas, including hope, optimism, happiness, honesty, patience, calmness, courage, loyalty, and wisdom. The PERMA model is applied to positive emotions, engagement, relationships, meaning, and accomplishment in couples. This model focuses on positive things such as good features, virtues, and human abilities. This can eventually lead to changes in lifestyle and attitudes (24) (Table I).

#### Intervention group 2

The second intervention group will receive IBCT counseling based on couple's therapy (21). In this method, couples are encouraged to have empathy and emotional acceptance in relationships, raise the threshold of tolerance in facing problems and improve problem-solving skills. This method teaches them to negotiate instead of judging, which can ultimately lead to positive changes in couples' behaviors (25) (Table II).

#### Intervention group 3 (control)

This group is considered an active control group. They will receive information regarding various infertility treatment techniques in ART (22, 26). Necessary care during treatment, the success and failure rate of ART, treatment complications, and cost of treatment will be discussed (Table III).

### Outcomes

The mean scores of the fertility problems will be determined (assessed by the FPI) in the women, men and couples at the baseline of the intervention, at the end of the 5 ${}^{\text{th}}$ wk of the intervention, and one month after the termination of the intervention (in the 9 ${}^{\text{th}}$ wk) in the three study groups. Also, the mean score of satisfaction with the counseling method will be determined (assessed by the satisfaction questionnaire) in women, men and couples at the end of the 5 ${}^{\text{th}}$ wk of the intervention, and in the 9 ${}^{\text{th}}$ wk in all three groups. Occurrence of pregnancy will considered as a secondary outcome, defined as a positive pregnancy test, which will be assessed in the follow-up in the 12 ${}^{\text{th}}$ wk.

### Data collection

The data will be collected by questionnaires at the baseline of the intervention, at the end of the 5 ${}^{\text{th}}$ wk of the intervention (the end of the intervention), and one month after the termination of the intervention (in the 9 ${}^{\text{th}}$ wk). Determination of pregnancy frequency and percentage will be carried out in the 12 ${}^{\text{th}}$ wk (Figure 1).

####  Data collection instruments 

Each husband and wife will complete the study questionnaires separately. All questionnaires in the baseline assessment will be completed face-to-face, but at the end of the 5 ${}^{\text{th}}$ wk of the intervention and at the 9 ${}^{\text{th}}$ wk, they will be completed electronically.

#### 2.11.1.1. Demographic questionnaire

Age, occupation, level of education, cause of infertility, duration of infertility, duration of the marriage, and duration of treatment will be assessed at the baseline of the intervention.

#### 2.11.1.2. FPI

The FPI questionnaire was developed by Newton in 1999 to determine stress associated with infertility. This scale includes 46 questions across five domains (social concern, sexual concern, relationship concern, rejection of parenthood, and need for parenthood). Its reliability has been reported to be 87% Cronbach's alpha. This questionnaire is a 6-point Likert scale, from strongly disagree = 1 to strongly agree = 6. Higher scores imply more fertility problems (27). It will be assessed at the baseline of the intervention, at the end of the 5 ${}^{\text{th}}$ wk of the intervention, and in the 9 ${}^{\text{th}}$ wk in the three study groups.

#### 2.11.1.3. Satisfaction with the counseling method scale

This variable will be measured by the researcher-made scale (Cronbach's alpha 0.87) (28). It scores between 1 (the least satisfaction) to 10 (the most satisfaction), using the visual analog scale method (29). It will be assessed at the end of the 5 ${}^{\text{th}}$ wk of the intervention and in the 9 ${}^{\text{th}}$ wk in the three study groups.

#### 2.11.1.4. Pregnancy

Pregnancywill be determined with one closed-ended question (yes or no) electronically in the 12 ${}^{\text{th}}$ wk.

**Table 1:** Content of the PERMA model intervention


**Sessions**	**Aim**	**Contents**
**1**	Positive emotions	The welcome session, explanation of rules and homework. Explanation of four key elements of emotional intelligence including management, perception, understanding, and using emotions regarding infertility. Happiness and life satisfaction are important subjects. Try to increase positive emotions about the past by forgiveness, about the present by physical pleasures and mindfulness, and about the future by hopefulness. Write down three things you experienced today that were fun and made you happy. Describe how you felt.
**2**	Engagement	Experience of flow in various activities by more concentration in the moment and increasing self-awareness. When time stops for you and you are completely absorbed by the task. Positive mental engagement and flow suppress mental ruminations regarding infertility. Write down three things you experienced today when all your attention was on something or someone and you were not aware of your surroundings. When the clock stops for you, when you lose consciousness, you are completely absorbed in your work. Describe how you feel.
**3**	Positive relationship	Positive and engagement are two elements to achieve well-being and positive relationships. Relationships are essential to well-being. More support and connectivity to others can promote a good purpose in life and meaning. This process is a valuable way to feel more positive. Kindness for others improves the feeling of well-being and increases capacity for love, compassion, empathy, teamwork, and cooperation. Positive relations involve participation and cooperation and so help us to avoid comparing ourselves with others, and promote comparing our diseases like infertility with couples who have children in an empathic way. Three things you experienced today where you had a positive experience with people. Only a very small part of being positive is related to isolation and loneliness. When was the last time you laughed out loud? When was the last time you felt indescribably happy? All of these happened alongside other people. By managing relationships and choosing the right people, we can have close and intimate relationships with those around us. Write down how you feel about these experiences. You can also practice kindness.
**4**	Meaning	Meaning is a feeling of belonging to the bigger community than the self by more socialization that produces a sense of meaning, such as through religion, family, and the community. Couples with infertility can create a better meaning of life with the help of other couples with infertility. Write down three things you experienced today that were important, significant, and meaningful to you personally. Write down how you feel about these experiences.
**5**	Accomplishment	Feeling of happiness regarding achievement, competence, and success in daily life. Review of gained positive things like having more support, empathy with another couple with infertility, successes in life or infertility treatments like the maturation of the oocyte, better sperm analysis test and things like these. Write down three things you experienced today when you were successful and thought you did a very good job and describe how you felt. Assessment of positive changes in thoughts, perceptions, beliefs, and emotions regarding fertility problems.
PERMA: Positive emotions, Engagement, Relationship, Meaning, Accomplishment		

**Table 2:** Content of IBCT intervention


**Sessions **	**Aims**	**Content**
**1**	Familiarity and evaluation	The welcome session, explanation of rules and homework. Establishing communication and carrying out initial evaluation based on functional analysis to achieve a codified formulation and treatment plan including theme, polarization, and mutual trap. Training skills regarding active hearing and concentration in a spousal relationship especially regarding infertility and fertility problems.
**2**	Feedback session	Familiarizing members with the treatment model, building trust and hope, and providing appropriate coordination between the focus on the current problems of the couples and the history of their marital relationship (the initial attraction that couples had for each other, etc.), examining the strengths of the marital relationship and also determining the compatible differences between them, the degree of commitment of the couples to the relationship, providing treatment formulation to the couple and setting goals.
**3**	Strategies for acceptance	Using empathetic joining and unified detachment methods to provide patterns for couples to experience their dissimilarities differently so that couple problems become a device for greater intimacy between them. Seeing fertility problems as a way for connecting the couple with more empathy.
**4**	Strategies for creating tolerance	Pointing to the positive aspects of negative behavior, practicing negative behaviors in a therapy session, as well as self-discipline to prevent couples from trying to change each other. Using behavior exchange techniques and teaching communication skills.
**5**	Strategies for making change	Using behavioral change techniques and communication, problem-solving skills training to make changes in couples' behavior directly. Avoidance of running away from fertility problems and spending more time which each other. Reviewing of positive changes in couple's relationship for better management of fertility problems using IBCT skills.
IBCT: Integrative-behavioral couple therapy		

**Table 3:** Content of infertility treatment training program


**Sessions**	**Aims**	**Content**
**1**	Introduce infertility and ART	Brief description regarding the definition of infertility and the causes of infertility which include both male and female factors. ART will be described. A brief explanation of IVF and ICSI etc. will be given to the couple. Treatment protocols will be explained. Necessary precautions will be described before the couple performs tests such as sperm analysis. Necessary training on exercise, physical activity, and diet will be given, if necessary.
**2**	Supplements and drugs	Guidelines for taking supplements in males and taking ovulation-inducing drugs before ART in females will be provided. Taking supplements and medications that physicians order will be described.
**3**	Treatment costs and drug side effects	Explanations about costs and insurance coverage, common problems that occur while taking medications, and side effects of medications.
**4**	Puncture of ovum, sperm processing and failure of treatment	The possibility of canceling treatment due to unsatisfactory female test results and sperm analysis will be explained. The probability of failure of treatment, sperm processing, and how to puncture the ovum will be provided in the form of educational videos via mobile phone.
**5**	Transfer method and post-ART care	Fertilization number of transferred embryos and transfer methods will be taught to couples through videos. Information on IVF failure and success rates per cycle and medication guidance for endometrial preparation for transmission will be provided. Post-ART care will be taught.
ART: Assisted reproductive technology, IVF: In vitro fertilization, ICSI: Intracytoplasmic sperm injection		

### Ethical considerations

The Ethics Committee of Shahid Sadoughi University of Medical Sciences approved this study with code IR.SSU.REC.1398.140.

### Statistical analysis

Frequency, percentage, mean, and standard deviation will be used to measure descriptive data. Shapiro-Wilk's normality test will be applied to determine the normal distribution of quantitative data. Chi-square test will be done for comparing qualitative data. Parametric analytic statistical tests like ANOVA will be applied for the measurement of the two primary outcomes (fertility problems and satisfaction with the intervention method) if the data have a normal distribution. P-values < 0.05 will be considered significant.

## 3. Discussion

Nowadays, many couples face the challenge of infertility and the problems resulting from its treatment. Infertility causes stress and anxiety in infertile couples which affect their lives. Psychological problems also exacerbate infertility. Women undergoing infertility treatment with in vitro fertilization or other methods may experience a change in their quality of life, marital relationship, and sexual functions. Psychological interventions have a significant impact on reducing these problems of infertile couples (30, 31). The PERMA model is used to increase well-being and health (13), increase happiness, improve depressive symptoms (12), and increase personality strengths (32). The effect of behavioral therapy on infertile couples is significant, which enriches communication, increases marital satisfaction, protects relationships, and helps to solve problems (17). In the first phase of the present study, the content of the interventions' sessions was developed and adjusted based on a literature review and the opinions of expert panel members from various related disciplines. The intervention sessions will be conducted by trained and experienced counselors in all three groups. These packages can be used for raising optimism, increasing communication skills, and providing infertility treatment information to couples and other researchers.

There are five building blocks in the PERMA model that enable flourishing and improved mood. We have chosen this model to assess whether improving the mood, after practicing the domains of the PERMA model, can lead to amelioration of tolerance in couples and the mean scores of the FPI.

In the IBCT model, couples learn communication skills, problem-solving skills, behavioral exchange techniques, and empathetic joining. The communication training program leads couples to communicate better and improve their conflict-resolution skills. We expect improvement in communication skills to lead to improvement of the mean scores of the FPI.

Many patients with infertility who have psychological stress do not receive mental health services. Couples seeking infertility therapy should be informed about the treatment process because a lack of information leads to stress. Perhaps using an infertility treatment training program can reduce their stress.

Although men, similarly to women, need psychological interventions, they are not usually supported by support services. Therefore, the present psycho-social services will be provided to infertile couples; not only women will be considered but also their spouses. Therefore, in the present study via using the content of the intervention packages and comparing the results in the three groups (women, men, and the couples), we hope that perceived infertility-related stress decreases. We also will compare the mean satisfaction scores of the three intervention methods in the women, men, and the couples. Besides enhancing the marital status by employing such methods, couples' satisfaction with these interventions may lead them to recommend these methods to other couples, or they may be interested in continuing these intervention methods in the future or applying these methods for other problems in their marital life. In addition, a combination of face-to-face and online methods may be useful in infertility treatment at the time of outbreaks of COVID-19 or other similar diseases that necessitate social distance regulations.

It is hypothesized that PERMA, IBCT, and infertility treatment training programs will help reduce stress reaction mechanisms in the present study. Reducing psychological problems can have positive effects on infertile couples, and according to previous studies, PERMA and IBCT methods can have positive effects on reducing stress in infertile couples. This study provides a package of a series of exercises for couples who can continue to use them after their couple's sessions. Participation of couples is one of the strengths of the present study. Another strength is that sessions are not held in groups - there is just one couple in each session. Thanks to the positive content of couple's therapy approaches and routine care, the needs, and problems of these couples can be reduced by using these models. Other strengths of this study include the involvement of a specialist team such as a reproductive health specialist and psychologist, a fellow in infertility and a couple's therapy consultant, as well as a midwifery consultant. Another strength is use of the FPI, which considers many aspects of fertility problems such as social concerns, sexual concerns, relationship concerns, rejection of a childfree lifestyle, and the need for parenthood.

We hypothesize that through this study we will help infertile couples to first acknowledge their infertility and then be able to reduce their fertility problems by better tolerating, coping with and dealing with these problems, and also gaining skills in solving problems, and seeing other positive aspects of their lives. The results of the present study might be used by the Health Ministry to develop policies towards considering the mental health of couples during infertility treatment. In addition, by increasing successful pregnancy rates through reducing stress, these interventions could lead to a decrease in the number of fertility treatments needed and so also reduced costs and other burdens.

Limitations of the present study include that, due to the nature of the interventions, it is impossible to blind the couples or counselors to the intervention allocations.

## 4. Conclusion

This study will assess social, sexual, relationship and parenthood concerns. A combination of online and face-to-face interventions is appropriate for times of COVID-19 outbreaks. Couple's counseling may provide better outcomes for fertility problems in comparison with group counseling. A package for intervention sessions will be provided and the effectiveness of two psychological interventions (PERMA and IBCT) will be compared with the control group. This study will try to optimize resilience during infertility treatment through learning better relationship and problem-solving skills, which may also have an indirect impact on pregnancy rate, burden of infertility, and costs of treatment due to increased effectiveness.

##  Conflict of Interest

All four authors declare that they have no conflict of interest.
